# Topochemical Reaction Induces Anisotropy, Decreasing Solid-State Thermal Conductivity

**DOI:** 10.1021/acsmaterialslett.5c01606

**Published:** 2026-01-19

**Authors:** Amalie Atassi, Sara Makarem, James F. Ponder, Alex H. Balzer, Joshua M. Rinehart, Shawn A. Gregory, Valentina Pirela, Jaime Martín, Patrick E. Hopkins, Natalie Stingelin, Shannon K. Yee

**Affiliations:** † School of Materials Science and Engineering, 1372 Georgia Institute of Technology, Atlanta, Georgia 30332, United States; ‡ Department of Materials Science and Engineering, 2358 University of Virginia, Charlottesville, Virginia 22904, United States; § George W. Woodruff School of Mechanical Engineering, 1372 Georgia Institute of Technology, Atlanta, Georgia 30332, United States; ∥ Materials and Manufacturing Directorate, Air Force Research Laboratory, Wright-Patterson AFB, Dayton, Ohio 45432, United States; ⊥ School of Chemical and Biomolecular Engineering, 1372 Georgia Institute of Technology, Atlanta, Georgia 30332, United States; # POLYMAT, Department of Polymers and Advanced Materials: Physics, Chemistry, and Technology and Faculty of Chemistry, 160665 University of the Basque Country UPV/EHU, Donostia-San Sebastián 20018, Spain; ∇ Universidade da Coruña, Campus Industrial de Ferrol, CITENI, Esteiro, 15403 Ferrol, Spain; ○ Department of Mechanical and Aerospace Engineering, 2358 University of Virginia, Charlottesville, Virginia 22904 United States; ● Department of Physics, 2358 University of Virginia, Charlottesville, Virginia 22904, United States

## Abstract

Realizing organic materials that exhibit a dynamic thermal conductivity requires a fundamental understanding of how molecular structure and processing affect thermal transport. Herein, we demonstrate that the photoinduced polymerization of [2,2′-bi-1*H*-indene]-1,1′-dione-3,3′-diheptylcarboxylate (BIT) into polyBIT results in over a 4-fold decrease in thermal conductivity as measured on polycrystalline thin-films in the through-plane direction, mostly perpendicular to the chain growth direction. Experimental determination of the material’s decreased heat capacity supports this view. Through theoretical calculations, we attribute this decrease in thermal conductivity in part to induced anisotropy in the polymer. We also discuss the non-negligible changes in morphology, phase transitions, and thermal degradation that serve to limit the thermal depolymerization reaction. This work highlights the different contributions one must consider when designing an organic thermal switch that operates in the solid-state.

## Graphical abstract

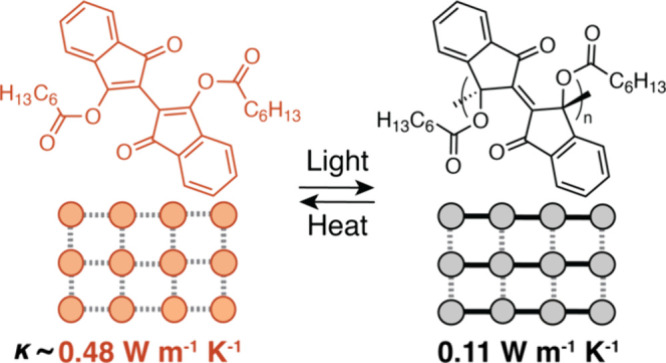

Identifying materials that dynamically control heat flow can actualize efficient thermal components such as thermal switches, regulators, and diodes.^1^ The materials utilized in these components can serve to scavenge waste heat,^2,3^ increase engine efficiencies, decrease power consumption, and generally improve the thermal performance of current energy management technologies.^1,4^ Organic materials offer the advantage of broad chemical versatility, which can enable the design of materials that undergo changes in chemical structure in response to external stimuli—an important criterion for developing thermal components.

Dynamic thermal transport in organic systems can be achieved when π-π stacking,^5,6^ cross-linking,^7^ and hydrogen^8,9^ interactions are reversibly altered. In each case, the extent of the bonding interactions is changed, whereby the strength of these interactions dictates the efficiency of thermal transport.^10^ The effect of bonding interactions on the material’s thermal conductivity (κ) is apparent in high-modulus polymeric fibers,^11^ for example, where κ is highest along the direction parallel to the molecularly aligned polymer chain (i.e., covalent bonding direction)^12,13^ and lower radial to the chain (i.e., secondary interactions direction).^14^

Topochemical polymerizations dynamically convert weak interactions into strong covalent bonds while maintaining a high degree of molecular order after the reaction. Notable changes in κ have been measured in topochemically reactive monomers, in which the magnitude of the change depends on chemical structure, interchain bonding, and chain alignment.^15–18^ Here, we focus on an organic small molecule, [2,2′-bi-1H-indene]-1,1′-dione-3,3′-diheptylcarboxylate (BIT; Figure 1a, left),^19^ that undergoes a topochemical polymerization to a polymer referred herein as polyBIT (Figure 1a, right). This material has been previously synthesized and shown to photopolymerize upon exposure to visible light as the external stimulus while maintaining its molecular order throughout the material,^19^ as schematically shown in Figure 1b. It has been demonstrated as a candidate for recyclable plastics,^20^ mechanical actuation,^21^ and photopatterning.^22^ Given its additional potential for thermal components, we synthesized BIT to examine the thermal transport properties. Details of the BIT synthesis (see Figure S1) and photopolymerization can be found in the Supporting Information.

**Figure 1:**
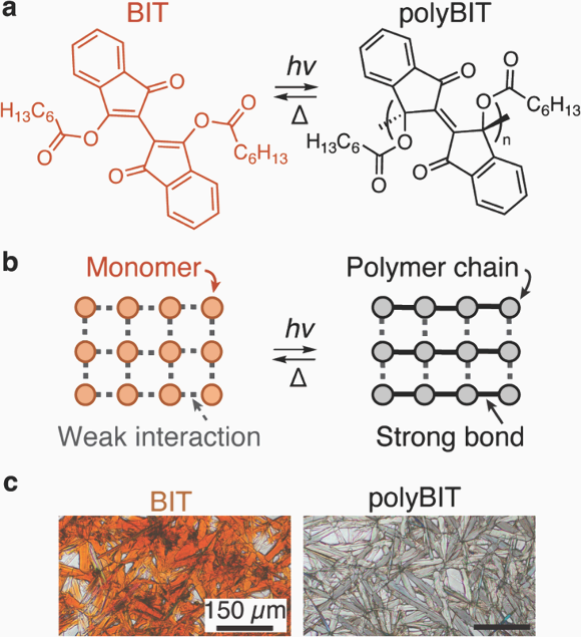
Topochemical polymerization of BIT into polyBIT. (a) Chemical structures of BIT (orange) and polyBIT (black). The forward polymerization reaction occurs when BIT is exposed to light. The reverse reaction, in principal, can be triggered with temperature.^19,20^ (b) Topochemical reactions occur in the crystalline state of BIT; hence, the molecular order and packing is largely preserved in the polymeric state.^20,23,24^ (c) Optical micrographs of BIT and polyBIT films illustrating their highly polycrystalline nature.

Upon exposure to visible light, the topochemical polymerization of BIT proceeds via homolytic cleavage of carbon–carbon double bonds in the α,β-unsaturated carbonyl functionality, resulting in the formation of new C–C double bonds between the two cyclopentyl rings of the monomer, and the consequent reduction of conjugation length. Hence, BIT absorbs light between approximately 360 to 580 nm and appears orange, whereas polyBIT does not and, thus, is visibly transparent as shown in Figure 1c. The different optical properties also allow straightforward monitoring of the reaction with ultraviolet–visible absorbance spectroscopy at multiple thicknesses (see absorbance spectra in Figure S2). Moreover, and as anticipated for a topochemical reaction, no drastic changes in solid-state structure and overall morphology are found after the polymerization. On the micrometer length scale, both BIT and polyBIT crystalline platelets ranging from 100 to 200 μm in length and approximately 20 μm in width are observed in optical microscopy (Figure 1c), with the primary difference being the previously noted change in absorbance profile and resulting color. More quantitatively, both materials feature well-defined grazing-incidence wide-angle X-ray scattering (GIWAXS) patterns, indicative of a high degree of molecular order^25^ (see Figure S3). Importantly, the patterns for BIT and polyBIT are similar in diffraction peak position and distribution with variances ranging from roughly 2–8% of the average peak position, confirming the topochemical reactivity of this system and corroborating previously reported crystallography data for these materials.^19,20^ Because these films are polycrystalline, small variations in the diffraction patterns and profiles are to be expected and attributed to changes in subpopulations after photopolymerization.

To examine κ, the various thermal contributions within the polycrystalline samples must be considered. A simplified schematic of three major thermal contributions, which are expected to manifest in a κ measurement for this system, is presented in Figure 2a. First, the organic crystallite will exhibit a κ whose change in magnitude upon photopolymerization depends on the direction of heat flow relative to the [0*k*0], (i.e., the direction of chain growth).^18,19^ Second, organic–organic and organic–metal interfaces are formed in these polycrystalline films that act as barriers to heat transport.^26–28^ Third, air voids (imaged in Figure 1c) are expected in the polycrystalline films that also will hinder heat transport because the κ of air is approximately 0.026 W m^–1^ K^–1^. We initially employed the 3ω method in a bidirectional geometry with a ∼60 μm wide heater line (see details in Section 3.1 in the Supporting Information) to measure κ of the organic material before and after photopolymerization.^29,30^ However, the large heater line width combined with the selection of a substrate with a higher κ than typical polymers (1.3 W m^–1^ K^–1^ for a-SiO_2_)^30–32^ resulted in a reduced measurement sensitivity of the organic crystallites (see Figure S4). With effective κ values of 0.20 ± 0.03 W m^–1^ K^–1^ for BIT and 0.15 ± 0.03 W m^–1^ K^–1^ for polyBIT, we posit that these measurements are most representative of the organic-metal interface rather than the organic crystallites.

**Figure 2:**
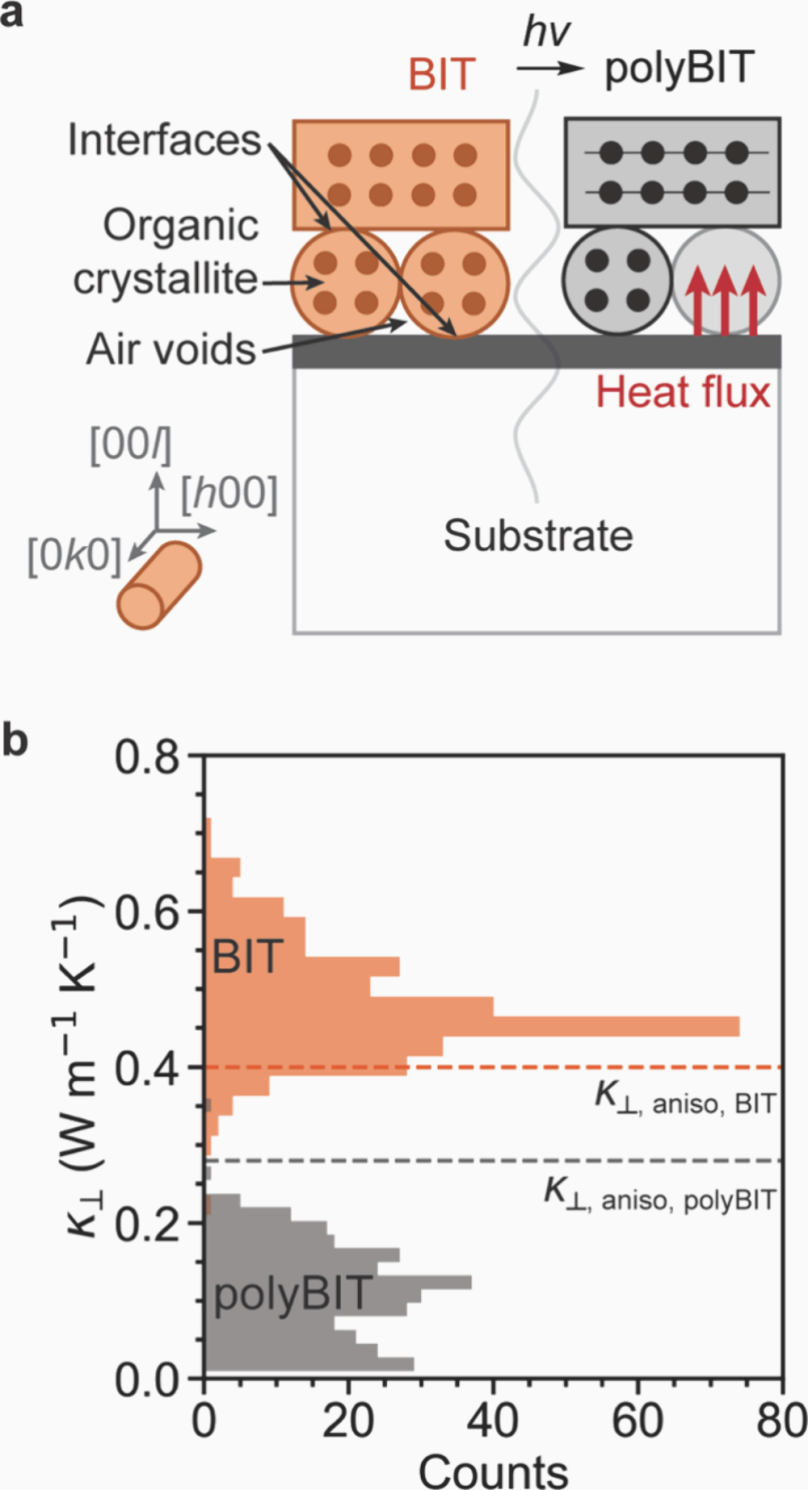
Thermal contributions and thermal conductivities of BIT and polyBIT in polycrystalline films. (a) Simplified side-view schematic of the polycrystalline film on top of an Al-coated a-SiO_2_ substrate, where the Al serves as the transducer for the time domain thermoreflectance measurement. A heating probe is focused through the a-SiO_2_ substrate onto the Al film, creating a heat flux that flows perpendicular to the direction of chain growth, *i.e*. the [0*k*0]. (b) Histogram of thermal conductivity measurements taken using time domain thermoreflectance by spatially scanning the pump and probe lasers across the polycrystalline film before and after polymerization.

Due to the spatial averaging of the 3ω method in this sample geometry, we also report on time-domain thermoreflectance (TDTR) to measure the thermal properties of individual crystallites.^33^ TDTR is an optothermal technique with a spatial resolution of 10 μm, i.e., sufficient to probe individual BIT and polyBIT crystallites (see Figure 1a and Section 3.2 in the Supporting Information for TDTR experimental details). Briefly, a laser beam is focused through the a-SiO_2_ substrate onto the metal transducer to take a local thermal measurement. The beam then scans across the entire sample (∼1 cm^2^), taking thermal measurements at each location. In Figure 2b, histograms of the κ distributions for BIT and polyBIT, obtained from TDTR, are presented. With this methodology, we found a decrease in κ after topochemical polymerization, from 0.48 ± 0.07 W m^–1^ K^–1^ for BIT to 0.11 ± 0.06 W m^–1^ K^–1^ for polyBIT. We acknowledge that some may favor the 3ω results over the TDTR results, or vice versa, and therefore we present both results, with each showing that the topochemical polymerization of BIT decreases the thermal conductivity. Specifically, the topochemical polymerization of BIT thus yields a thermal contrast of 4.4, which for context is notably higher than that of solid-to-melt transitions of water (*r* ≈ 3.5 at 0 °C)^30^ and wax (*r* ≈ 2–3),^34^ two common materials used for thermal management and mechanically actuated thermal switching.

This measured change in κ is in part due to the anisotropy induced by chain growth along the [0*k*0] direction after photopolymerization. The extent of its effect on κ was quantified with an anisotropic thermal conductivity (κ_aniso_) proposed by Chen and Dames.^35^ For chain-like materials in the high temperature limit, the κ_aniso_ measured perpendicular to the polymer chain is

$$\kappa_{\mathrm{aniso},⊥}=\frac{k_{B}k_{⊥}^{2}}{8\pi }\frac{v_{⊥}^{2}}{v_{∥}}$$ 1

where *k*_⊥_ is the perpendicular wavevector cutoff, and *v* is the average speed of sound in the perpendicular (⊥) and parallel (∥) orientations. This model predicts the thermal conductivity perpendicular to the chain growth direction to decrease after photopolymerization by 30%, from 0.40 to 0.28 W m^–1^ K^–1^, plotted as dashed lines in Figure 2b. The anisotropic model more accurately reflects the decrease in κ after photopolymerization than the minimum thermal conductivity (i.e., Cahill-Pohl model), which erroneously calculates an approximately 17% increase in κ after photopolymerization.^36^ If κ were instead measured in plane, along the direction of chain growth, κ_aniso_ should increase 3.6× due to the conversion of weak intermolecular interactions into strong covalent bonds. A detailed discussion on the values and assumptions used to model κ can be found in Section 3.3 in the Supporting Information. Additionally, we considered numerous effective medium models but found them to be inconclusive, found in Section 3.4 in the Supporting Information.

However, anisotropy does not fully explain the 77% measured decrease or 4.4× change in κ, suggesting other factors are also responsible. Because κ is linearly proportional to a material’s heat capacity at constant pressure (*c*_p_), an indicator of both the degrees of freedom in the material and the amount of energy it can absorb before its temperature increases, we examined *c*_p_ using temperature-modulated differential scanning calorimetry (experimental details in Section 4.1 in the Supporting Information). At 27 °C, *c*_p_ is 1.13 ± 0.05 J g^–1^ K^–1^ for BIT compared to 1.04 ± 0.04 J g^–1^ K^–1^ for polyBIT, as shown in Figure 3, which corresponds to an 8% decrease—similar to other polymerization reactions.^37,38^ With increasing temperature, however, the difference in *c*_p_ between BIT and polyBIT increases. Subsequent calorimetry experiments identified this difference is due to an appreciable latent heat contribution corresponding to a solid–solid phase transition in BIT (also imaged with optical microscopy, see Figure S8). Unfortunately, increasing the temperature above this phase transition in BIT results in some mass loss (see Figure S9) and limits the mass yield of the reverse thermal depolymerization reaction in polyBIT due to local overheating (see discussion in Section 4 in the Supporting Information).^20^ While Dou and co-workers circumvented this in their polycrystalline films by recuperating the depolymerized material in a solvent bath,^20^ this solution is impractical for solid-state thermal switching.

**Figure 3:**
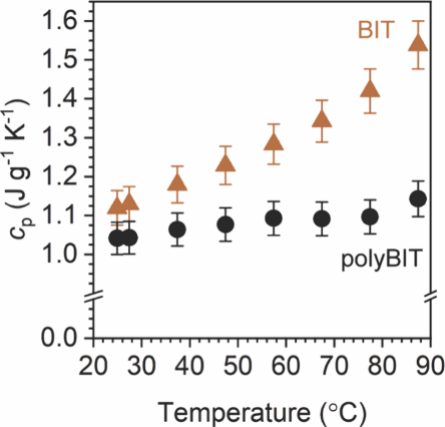
Temperature-dependent heat capacity for BIT and polyBIT films. (a) Effective thermal conductivity (solid bars) using the bidirectional 3ω and heat capacity at constant pressure (hatched bars) measured using temperature-modulated DSC for polyBIT and BIT at room temperature. (b) Thermal conductivity of polyBIT and BIT polycrystalline films measured using time-domain thermoreflectance.

We conclude that the topochemical polymerization of BIT leads to a high thermal contrast compared to other organic systems that function based on a liquid–solid phase transition.^5,34^ Specifically, the κ of BIT was demonstrated to decrease upon topochemically reacting into polyBIT after exposure to light, resulting in a 4.4× change. This thermal contrast is higher than that for solid–liquid transitions of H_2_O (3.5×)^30^ and waxes (2–3×)^34^ and is comparable to those achieved in mercury (4×).^39^ While this change does not outperform currently used thermal switches based on mechanical contact actuation (100×),^40^ for example, it is significant compared to other thermal switches based on solid–liquid phase transitions (3.5×),^5^ especially when considering that the reaction from BIT to polyBIT occurs entirely in the solid-state. We determined the decrease in κ after photopolymerization is primarily attributed to induced anisotropy in the direction perpendicular to chain growth as well as the decrease in *c*_p_. Potential changes in the material’s density and crystallite subpopulations also may affect the thermal resistances in the metal–organic, organic–organic, and organic-air interfaces, further contributing to the magnitude change in κ. Lastly, we note that the reverse solid-state reaction from polyBIT to BIT is possible but limited by local overheating in the polycrystalline film. Thus, special consideration should be taken when processing this material for thermal switching applications. In identifying the thermal contributions observed in this organic system, we hope this work motivates further investigations toward engineering solid-state organic thermal switches.

## Supplementary Material



## Notes

Synthetic, processing, instrumentation, and analysis details, along with additional figures and tables (PDF)
