# Supplementary material for: Topochemical Reaction Induces Anisotropy, Decreasing Solid-State Thermal Conductivity
Source: ACS Mater Lett. 2026 Jan 19;8(2):646–50. doi: 10.1021/acsmaterialslett.5c01606 (PMC12869699; doi:10.1021/acsmaterialslett.5c01606)
Supplement: Supplementary file 1 [file tz5c01606_si_001.pdf]

Supplementary Information

Topochemical reaction induces anisotropy, decreasing solid-state thermal conductivity

Amalie Atassi,[1] Sara Makarem,[2] James F. Ponder Jr.,[3,4] Alex H. Balzer,[5] Joshua M. Rinehart,[1] Shawn A. Gregory,[1] Valentina Pirela,[6] Jaime Martín,[6,7] Patrick E. Hopkins,[2,8,9] Natalie Stingelin,[1,5] Shannon K. Yee.[*3]

1. School of Materials Science and Engineering, Georgia Institute of Technology, Atlanta, GA 30332, USA.
2. Department of Materials Science and Engineering, University of Virginia, Charlottesville, VA 22904, USA.
3. George W. Woodruff School of Mechanical Engineering, Georgia Institute of Technology, Atlanta, GA 30332, USA. E-mail: shannon.yee@me.gatech.edu
4. Materials and Manufacturing Directorate, Air Force Research Laboratory, Wright-Patterson AFB, Ohio 45433, United States; AV, Dayton, Ohio 45432, United States
5. School of Chemical and Biomolecular Engineering, Georgia Institute of Technology, Atlanta, GA 30332, USA.
6. POLYMAT, Department of Polymers and Advanced Materials: Physics, Chemistry, and Technology and Faculty of Chemistry, University of the Basque Country UPV/EHU, Donostia-San Sebastián 20018, Spain.
7. Universidade da Coruña, Campus Industrial de Ferrol, CITENI, Esteiro 15403, Ferrol, Spain.
8. Department of Mechanical and Aerospace Engineering, University of Virginia, Charlottesville, VA 22904, USA.
9. Department of Physics, University of Virginia, Charlottesville, Virginia 22904, USA

Table of Content

1.1. Monomer synthesis. 3
1.2. Film fabrication. 3
1.3. Photo-polymerization. 3
2. Optical and structural characterization 4
2.1. Ultraviolet-visible absorption spectroscopy. 4
2.2. Optical microscopy. 4
2.3. Grazing incidence wide angle X-ray scattering. 4
3. Thermal conductivity measurements and analysis 6
3.1. Bidirectional 3ω measurements 6
3.2. Time domain thermoreflectance measurements 7
3.3. Isotropic versus anisotropic thermal conductivity models 9
4. Thermophysical characterization 13
4.1. Differential scanning calorimety and heat capacity at constant pressure measurements. 13
4.3. Thermogravimetric analysis. 14
5. References 15

1. Synthesis, sample preparation, and photo-polymerization

1.1. Monomer synthesis. Monomer was synthesized following procedures report by Dou et *al* and was confirmed by NMR, as seen in **Figure S1**.[1]

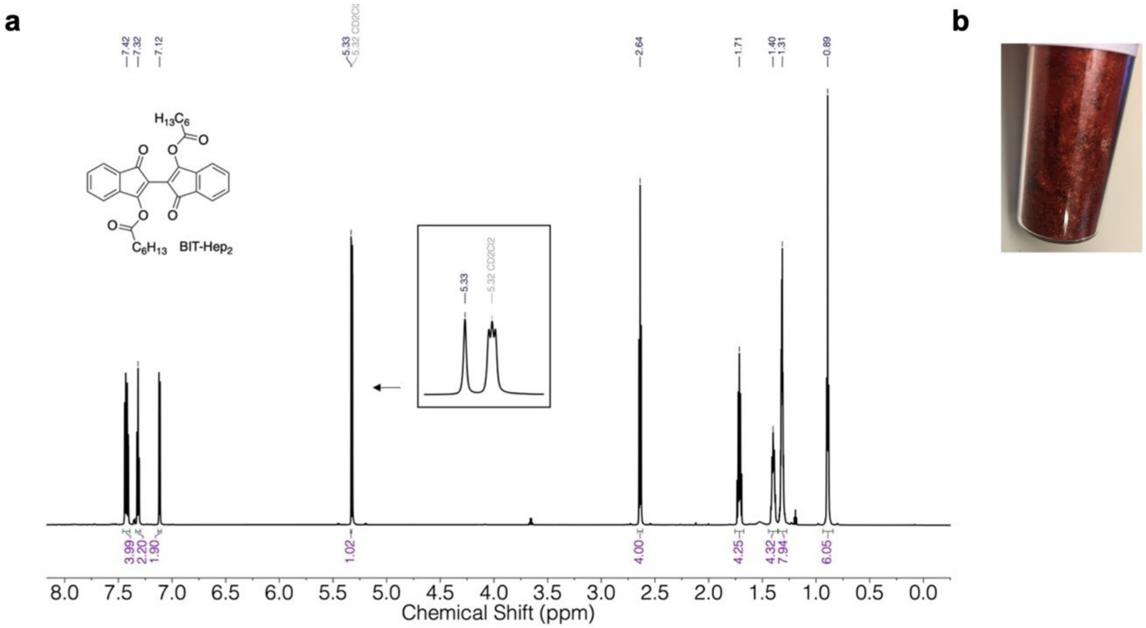

Figure S1: Synthesis of BIT. a) ^{1}H-NMR (700 MHz, 64 scans) of the BIT monomer in DCM-D_2. Spectrum is consistent with previous report.[1] b) Photograph of synthesized monomer powder in a vial.

1.2. Film fabrication. Thin films approximately 60 nm thick were made by spin-casting a 15 mg/mL solution of BIT in chloroform, consistent with Dou et *al.*'s procedure.[1] Thick films between 10-100 μm thick were melt-casted at 100 °C to melt the monomer powder. The thickness of each film was measured with a Bruker Dektak stylus profilometry. A K Control Coater was used to wire-bar the viscous liquid across the substrates, either glass, Au-patterned glass, or Al-coated glass. Glass substrates were composed primarily of amorphous silicon dioxide (a-SiO_2). The K-Bar had a wire width of 0.3 mm, was positioned ~0.05 mm above the substrate, and was drawn at the slowest speed of 2 m/min. Finally, the film was cooled slowly by turning off the heated stage and allowing the film's temperature to equilibrate with ambient.

1.3. Photo-polymerization. A high-pressure sodium lamp with a power of 600 W was used to polymerize monomer films. The time needed to fully polymerize the film is dependent on the film's thickness and was confirmed using ultraviolet-visible absorption spectroscopy (see SI **Section 2.1.**). Thin films required 15 minutes to fully polymerize, and thick films needed at least 4 hours of exposure to light.

2. Optical and structural characterization

2.1. Ultraviolet-visible absorption spectroscopy. Absorbance of thin and thick films were measured in transmission mode using an Avantes Avaspec-ULS2048CL-EVO-RS optic spectrometer equipped with a Mikropack DH-2000 deuterium-tungsten, halogen light source.

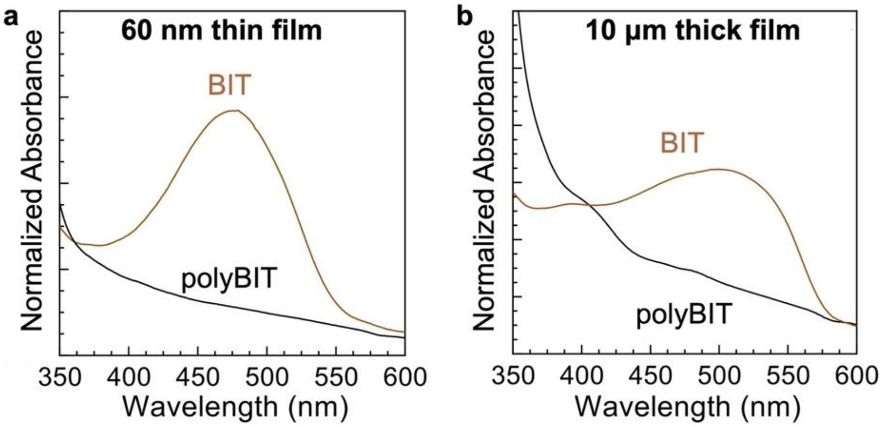

Figure S2: UV-visible absorbance of (a) thin, spin-casted monomer and polymer films normalized to 360 nm and (b) thick, melt-casted monomer and polymer films normalized to 410 nm. In both thin and thick films, the absorption band from 400-550 nm decreases, indicative that the polymerization occurs. We note that the absorbance values for the thick monomer and polymer films were on the order of 1, indicating that most of the incident signal was not transmitted. Therefore, the spectra of the thin film is most representative of the organic material's optoelectronic structure.

2.2. Optical microscopy. Micrographs of the monomer and polymer films were captured using a DM 2700P Leica microscope in transmission mode. Micrographs of the monomer and polymer films on 3ω devices were captured using a ToupCam camera.

2.3. Grazing incidence wide angle X-ray scattering. Grazing incidence wide-angle X-ray scattering (GIWAXS) measurements were performed at the BL11 NCD-SWEET at ALBA Synchrotron Radiation Facility (Barcelona, Spain). The incident X-ray beam energy using a channel cut Si (1 1 1) monochromator was set to 12.4 eV. 2D GIWAXS patterns were corrected as a function of the components of the scattering vector (q). The angle of incidence α_i was set between 0.1° and 0.2° to ensure surface sensitivity. The data is expressed as a function of the scattering vector, which was calibrated using Cr_2O_3, obtaining a sample-to-detector distance of 201.17 mm. The scattering patterns were recorded using a Rayonix LX255-HS area detector, which consists of a pixel array of 1920 × 5760 pixels (H × V) with a pixel size of 44 × 44 μm^2.

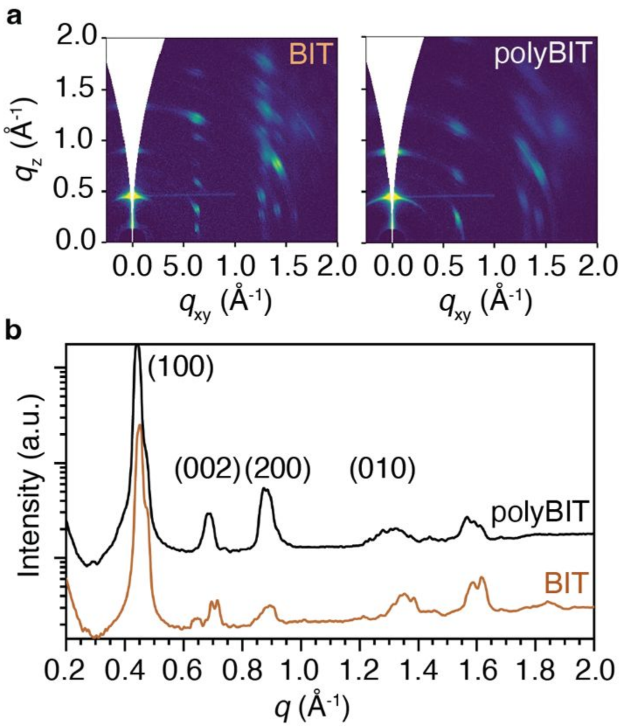

Figure S3: Two-dimensional grazing-incidence wide-angle X-ray scattering patterns of BIT and polyBIT thin films, and (c) corresponding radially integrated q-space patterns.

Table S1: Summary of average and full-width at half maximum variance in *d*-spacings for BIT and polyBIT. The scattering planes were identified based on previously published crystallography for these materials.[1]

**Plane**	**d_{hkl} BIT (Å)**	**d_{hkl} polyBIT (Å)**
(100)	4.6 ± 0.31	4.5 ± 0.31
(002)	6.8 ± 0.52	6.9 ± 0.18
(200)	8.8 ± 0.41	8.8 ± 0.28
(010)	13.1 ± 0.46	13.1 ± 0.57
(005)	16.0 ± 0.39	15.9 ± 0.39

3. Thermal conductivity measurements and analysis

3.1. Bidirectional 3ω measurements. The effective thermal conductivity, κ_{eff}, of thick BIT films before and after photo-polymerization were measured using the bidirectional 3ω method, as shown in **Figure S3a**.[2] First, a gold heater line with a known temperature coefficient of resistance is deposited on a substrate using thermal evaporation and a patterned photomask. The substrate must have comparable thicknesses and thermophysical properties to the sample, such as a-SiO_2 with a κ ~1.3 W $m^{-1}K^{-1}$ and C_v~1.6 J cm^{-3} K^{-1},[3-7] such that heat flows radially in two directions—into the film of interest and into the substrate.[2] Then, a thick film is melt-casted on top of the heater line and substrate.

The heater line half-width (b) must be smaller than the thermal penetration depth (L_p) (b/L_p < 5), and the substrate and film thicknesses (d_s and d_F) must be considered semi-infinite (d/L_p > 2).[8, 9] After these assumptions are met, the measurement is conducted. An alternating current with a frequency of 1ω is applied to the heater line, inducing local Joule heating in the sample with a frequency of 2ω. This Joule heating will cause a temperature change (ΔT) that depends on the electrical resistance of the metal heater line. This resulting change in resistance is measured as the heater line's voltage response, with a frequency component at 3ω ($V_{3\omega}$), and proportional to ΔT.[8] The ΔT of BIT before and after photo-polymerization are measured as a function of frequency (f). κ_{eff} is extracted from the temperature response, ΔT, which is equal to the amplitude of the Joule heating power times a thermal transfer function, or thermal impedance, Z. **Figure S3b** shows that the temperature response as a function of frequency is well modeled by the real component of the thermal transfer function, which can be thought of as a thermal impedance and is only a function of geometry and the thermophysical sample properties. The linear fit is equal to the real component of the thermal impedance, Z:

$$Z(\omega) = \frac{1}{2\pi\kappa L}\left[-ln\omega + \ln\left(\frac{\alpha}{b^2}\right) + \eta\right]$$

where L is heater line length, α is the thermal diffusivity, and η is a goodness of fit parameter that usually results in a value greater than 0.8 if the above assumptions are satisfied.[10] To calculate the κ_{eff} of the monomer and the polymer, the thermal conductivity of the substrate, κ_{sub}, is measured. Then, the thermal conductivity of the thick film on top of the substrate is measured. Finally, the effective thermal conductivity for BIT before and after polymerization is calculated by subtracting the measured thermal conductivity of the substrate from the measured thermal conductivity of the film on the substrate.[2] **Figures S3c-d** present the sensitivity analyses for the bidirectional geometry. Three separate samples were each measured before and after photo-polymerization.

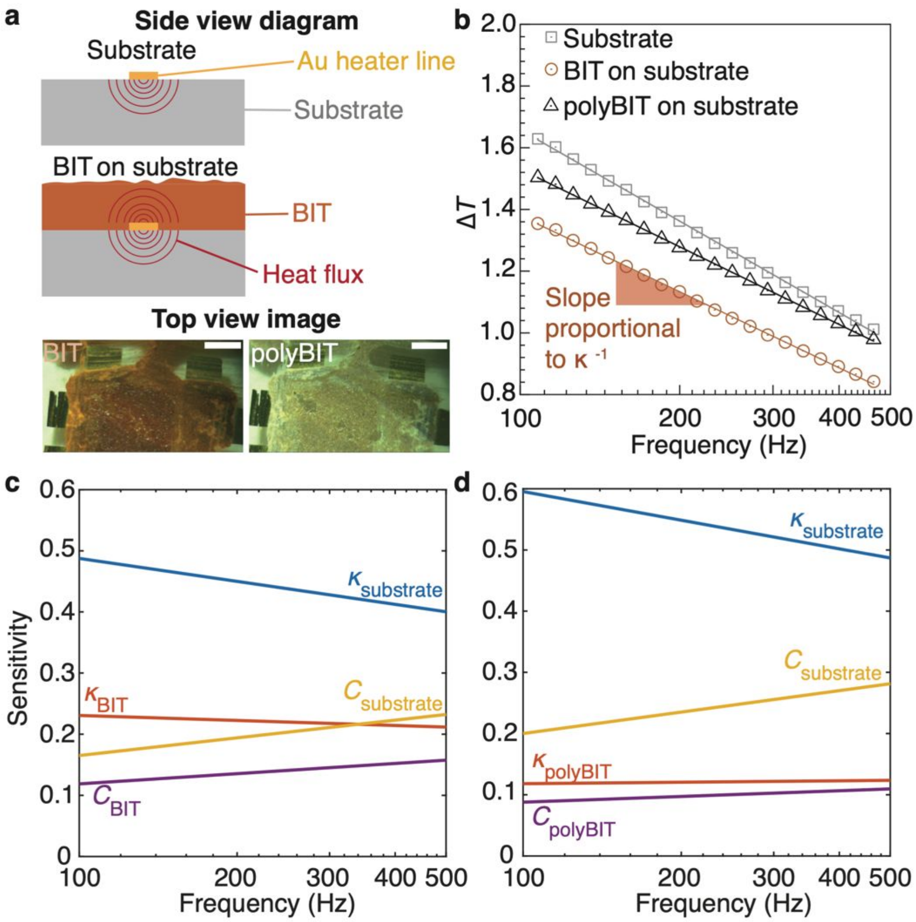

Figure S4: The 3ω technique in a bidirectional architecture can be used to measure polycrystalline organic material. (a) Side view diagram of the bidirectional geometry for 3ω technique[2] and top view optical image of the BIT polycrystalline film before and after photo-polymerization. The scale bar length in the top view images is equal to 1.5 mm. (b) Example of temperature response of the substrate, BIT on the substrate, and polyBIT on the substrate. (c) Sensitivity analysis of a bidirectional slope model for BIT and (d) polyBIT. Here, we show that while the measurement is more sensitive to the thermophysical properties of the glass substrate, a change in the slope of ΔT *vs.* log(f) is still recorded after the film is deposited on top, indicating more thermal conduction out of the heater line.

3.2. Time domain thermoreflectance measurements. The thermal conductivity, κ, of thick BIT films before and after photo-polymerization were measured in a bidirectional geometry using time domain thermoreflectance. An aluminum transducer layer (thickness ~80 nm) is deposited on a-SiO_2, an optically transparent substrate, using an e-beam evaporator. Al is chosen because of its large thermo-reflectance coefficient at a wavelength of 808 nm,[11] which facilitates the detection of extremely minute changes in thermo-reflectance and enables better signal-to-noise ratios. Thick BIT films are melt-cast onto the Al transducer. A Ti:sapphire Spectra Physics Tsunami laser (80 MHz pulse repetition rate, 11.5 nm FWHM, at an 808 nm center wavelength) is used. The output from the Tsunami oscillator is split into a pump and probe beam, with the spot sizes focused down to a $1/e^2$ radii of 9.8 μm and 5.6 μm, respectively. Because the melt-cast process produces films

on the order of several 10s μm, the films were analyzed as semi-infinite layers relative to the thermal penetration depth.

The details of this measurement technique and the thermal model that relates the experimental data to thermal properties are given elsewhere.[12, 13] Briefly, an electro-optically modulated pump beam with a modulation frequency of 8.8 MHz is focused through the transparent SiO_2 substrate onto the Al metal transducer film. The beam locally heats the Al surface, and this heat travels bidirectionally into the substrate and into the film of interest. The metal transducer's refractive index changes as a function of temperature. A mechanically delayed probe beam with a time domain of 5.5 ns monitors the change in the metal's thermoreflectance. A lock-in amplifier demodulates the signal and provides amplitude and phase data as a function of pump-probe delay time in the form of a thermoreflectance decay signal. This signal is strongly sensitive to the thermal effusivity, e, of the film and substrate, where $e = \sqrt{\kappa\, c_p\, \rho}$, and c_p is the specific heat capacity and ρ is the density. The thermal effusivity parameters for SiO_2 are known,[14] and the thermal effusivity parameters for BIT and polyBIT are measured in this study and previously.[1] The density of BIT and polyBIT changes by less than 5% as measured in this study using GIWAXS and previously reported using X-ray crystallography to be 1.29 g cm^{-3} and 1.34 g cm^{-3} for BIT and polyBIT.[1] Therefore, the volumetric heat capacities of BIT and polyBIT are 1.46×10^6 and 1.40×10^6 J m^{-3} K^{-1}. With these parameters, the decay curves are then fit with a heat conduction model to obtain the thermal properties of the sample.[12, 13]Assuming an isotropic κ model, the κ of the sample can be uniquely fit with a total systematic uncertainty of 10%, similar to previous work.[15]

To acquire a statistically meaningful representation of the full heterogeneity of the polycrystalline films, the pump and probe beams were scanned across a 2 by 2 mm^2 area of the sample with uniform spacing, to collect a raster of approximately 400 individual measurements.

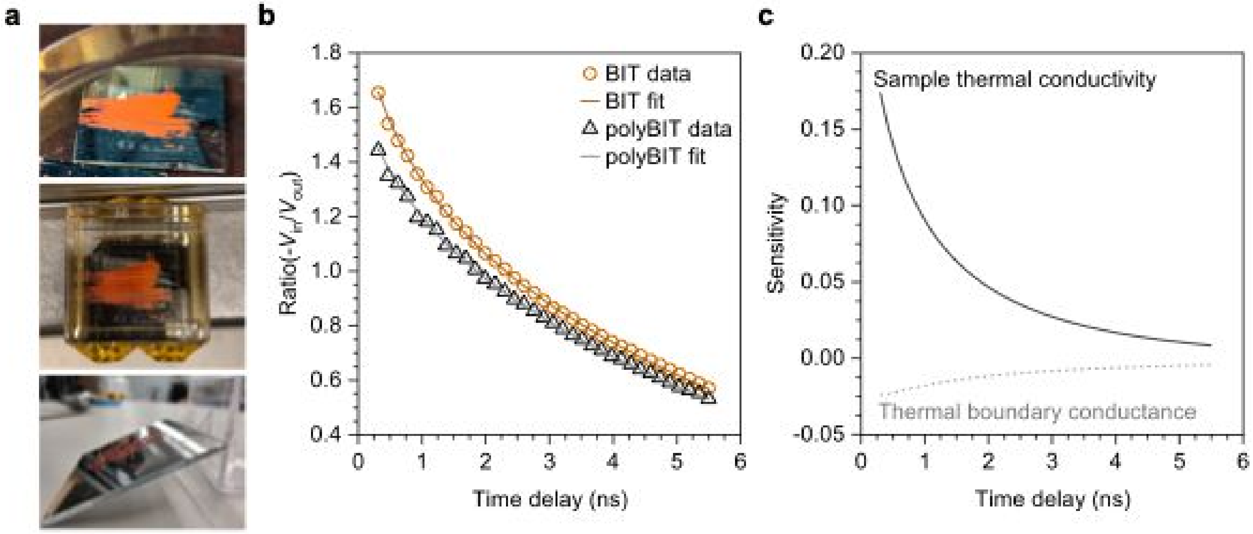

Figure S5: TDTR experimental results and sensitivity analysis. a) Photos of TDTR monomer films. b) Experimental results of a typical TDTR measurement. The ratio of the measured signal's in-phase (V_{in}) and out-of-phase (V_{out}) components as a function of time delay are fit to extract the thermal conductivity for BIT and polyBIT. c) Measurement sensitivity to the sample's thermal conductivity and the thermal boundary conductance between the film and metal transducer as a function of time delay.

3.3. Isotropic versus anisotropic thermal conductivity models. The isotropic thermal conductivity, κ_{iso}, was calculated for BIT and polyBIT using the equation prescribed by Cahill and Pohl:[16]

$$\kappa_{\mathrm{iso}}=\left(\frac{\pi}{48}\right)^{\frac{1}{3}} k_B\, n^{\frac{2}{3}}\,(v_l+2v_t)$$

where k_B is the Boltzmann constant, n is the effective number density of oscillators, v_l is the longitudinal speed of sound, and v_t is the transverse speed of sound. Since not all vibrational modes may be active at room temperature, n was calculated using the following set of rules previously published to more accurately reflect the oscillator density:[17, 18]

1. Each H atom contributes 0 vibrational modes.
2. Each C, N, or O atom loses 0.5 vibrational modes for each chemical bond with atoms (excluding H).
3. Each benzene ring is treated as two effective atoms and has 6 vibrational modes.
4. The effective oscillator density is the density of total vibrational modes divided by 3.

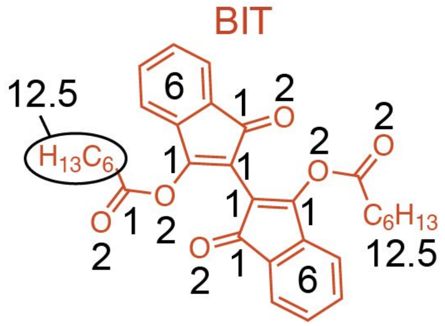

Figure S6: Vibrational modes of each functional group for BIT using the above-mentioned rules. The total number of vibrational modes is 57 for both BIT and polyBIT since the topochemical polymerization only rearranges bonds.

The below equation was used to calculate n:

$$n=\frac{57\,\rho\, N_A}{3\, M_0}$$

where ρ is the density, N_A is Avogadro's number, and M_0 is the molecular weight of the repeat unit. Previously published ρ values determined from crystallography were used.

To calculate v_l and v_t, the ρ, elastic modulus (E), and Poisson's ratio (ν) of BIT and polyBIT must be known. The elastic moduli perpendicular ($E_\perp$) to the crystal length and polymer chain were taken from published nanoindentation measurements on single crystals.[19] The elastic moduli parallel ($E_\parallel$) to the chain direction, *i.e.* along the [0*k*0], for polyBIT was taken from published tensile testing[1] and indicate $E_{\parallel,\mathrm{polyBIT}} \approx 3E_{\perp,\mathrm{polyBIT}}$, consistent with anisotropic measurements on high modulus fibers.[20] However, we must note that the reported $E_{\parallel,\mathrm{polyBIT}}$ may be an underestimation since the fibers were prone to slip fractures during the measurement. Moreover, $E_{\parallel,\mathrm{BIT}}$ was not measured.[1] Nonetheless, we assert that BIT should exhibit more isotropic properties than polyBIT, so $E_{\parallel,\mathrm{BIT}} = E_{\perp,\mathrm{BIT}}$. Next, we use ρ from published crystallography.[1] Lastly, we must assign a $\nu = 0.35 - 0.5$, where 0.5 is that of an incompressible solid. While tensile testing of some topochemical crystals suggest a $\nu \approx 0.5$,[21] this value would indicate these materials are

incompressible and yields κ_{iso} values an order of magnitude higher than that of typical organics. Other structural factors may be at play such that v may be closer to that of other organic materials at around 0.35, the value used for our calculations.[18, 21]

The anisotropic thermal conductivities, κ_{aniso}, parallel and perpendicular to the crystal length/polymer chain were calculated for BIT and polyBIT using the following equation presented by Chen and Dames for chain-like materials in the high-temperature regime:[22]

$$\kappa_{aniso,\parallel} = \frac{k_B k_\perp^2}{4\pi} \ln\left(\frac{2\theta_{D,\parallel}}{\theta_{D,\perp}}\right) \text{ and } \kappa_{aniso,\perp} = \frac{k_B k_\perp^2 v_\perp^2}{8\pi \ v_\parallel}$$

where k is the wavevector cutoff, θ_D is the Debye temperature, and v is the average speed of sound approximately equal to $(v_l+2v_t)/3$ for each direction.[23]A key assumption of this anisotropic model is that a material's anisotropic phonon dispersion can be modeled by the anisotropic Debye dispersion, which has ellipsoidal iso-energy surfaces in k-space.[24] Then, the wavevector cutoffs are fixed to the effective oscillator density calculated above:[22, 24]

$$n = 2.88 \times 10^{28} = \frac{1}{6\pi^2} k_\perp^2 k_\parallel$$

The wavevector cutoffs must be further constrained, which can be done by assigning the values from the first Brillouin zone along the corresponding crystallographic directions. Here, we apply the same ratio of $k_\perp / k_\parallel = 4.56$ previously reported by Chen and Dames.[22] **Table S2** summarizes the thermal conductivities.

Table S2: Summary of thermal conductivity values considered isotropically (κ_{iso}) or anisotropically in the direction perpendicular ($\kappa_{aniso,\perp}$) and parallel ($\kappa_{aniso,\parallel}$) to the polymer chain.

Property	BIT	polyBIT	Δ (%)
κ_{iso} (W m^{-1} K^{-1})	0.29	0.34	+17
$\kappa_{aniso,\perp}$ (W m^{-1} K^{-1})	0.40	0.28	– 30
$\kappa_{aniso,\parallel}$ (W m^{-1} K^{-1})	0.56	2.00	+257

3.4 Effective medium analysis

To help understand the 3ω and TDTR measurements, seven two-component models were examined, where the two components are the organic material, BIT or polyBIT, and air. **Table S3** lists the name and formula of each equation plotted in **Figure S7**. To provide further guidance for this analysis, ImageJ was employed to estimate volume fractions, but the volume of void/air is a free parameter.

Table S3: Tabulated equations used in effective medium analysis. κ_{eff} is effective thermal conductivity, κ_{c} is thermal conductivity of continuous phase, κ_{d} is thermal conductivity of dispersed phase, κ_{air} is thermal conductivity of air = 0.026 W m^{-1} K^{-1}, V_{air} is volume fraction of air, κ_{BIT} is thermal conductivity of BIT before (0.54 W m^{-1} K^{-1}) or polyBIT after (0.17 W m^{-1} K^{-1}) photo-polymerization, a is the void radius equal to 50 μm, and h_{c} is the interface conductivity equal to 5 W m^{-2} K^{-1}

**No.**	**Model**	**Equation**
(1)	Parallel	$\kappa_{\mathrm{eff}} = (1 - V_{air})/\kappa_{BIT} + V_{air}/\kappa_{air}$
(2)	Maxwell-Eucken, $\kappa_{\mathrm{c}} > \kappa_{\mathrm{d}}$	$\kappa_{\mathrm{eff}} = \kappa_{BIT} \frac{2\kappa_{BIT} + \kappa_{air} - 2(\kappa_{BIT} - \kappa_{air})V_{air}}{2\kappa_{BIT} + \kappa_{air} + (\kappa_{BIT} - \kappa_{air})V_{air}}$
(3)	Hasselman-Johnson, spherical	$\kappa_{\mathrm{eff}} = \kappa_{BIT} \frac{\left[2\left(\frac{\kappa_{air}}{\kappa_{BIT}} - \frac{\kappa_{air}}{ah_c} - 1\right)V_{air} + \frac{\kappa_{air}}{\kappa_{BIT}} + \frac{\kappa_{air}}{ah_c} + 2\right]}{\left[\left(1 - \frac{\kappa_{air}}{\kappa_{BIT}} + \frac{\kappa_{air}}{ah_c}\right)V_{air} + \frac{\kappa_{air}}{\kappa_{BIT}} + \frac{\kappa_{air}}{ah_c} + 2\right]}$
(4)	Effective medium theory	$\kappa_{\mathrm{eff}} = \frac{1}{4}\Big((3V_{air} - 1)\kappa_{air} + [3(1 - V_{air}) - 1]\kappa_{BIT} + \sqrt{[(3V_{air} - 1)\kappa_{air} + (3(1 - V_{air}) - 1)\kappa_{BIT}]^2 +}$
(5)	Hasselman-Johnson, cylindrical	$\kappa_{\mathrm{eff}} = \kappa_{BIT} \frac{\left[\left(\frac{\kappa_{air}}{\kappa_{BIT}} - \frac{\kappa_{air}}{ah_c} - 1\right)V_{air} + \left(1 + \frac{\kappa_{air}}{\kappa_{BIT}} + \frac{\kappa_{air}}{ah_c}\right)\right]}{\left[\left(1 - \frac{\kappa_{air}}{\kappa_{BIT}} + \frac{\kappa_{air}}{ah_c}\right)V_{air} + \left(1 - \frac{\kappa_{air}}{\kappa_{BIT}} + \frac{\kappa_{air}}{ah_c}\right)\right]}$
(6)	Maxwell-Eucken, $\kappa_{\mathrm{c}} < \kappa_{\mathrm{d}}$	$\kappa_{\mathrm{eff}} = \kappa_{air} \frac{2\kappa_{air} + \kappa_{BIT} - 2(\kappa_{air} - \kappa_{BIT})(1 - V_{air})}{2\kappa_{air} + \kappa_{BIT} + (\kappa_{air} - \kappa_{BIT})(1 - V_{air})}$
(7)	Series	$\kappa_{\mathrm{eff}} = \frac{1}{(1 - V_{air})/\kappa_{BIT} + V_{air}/\kappa_{air}}$

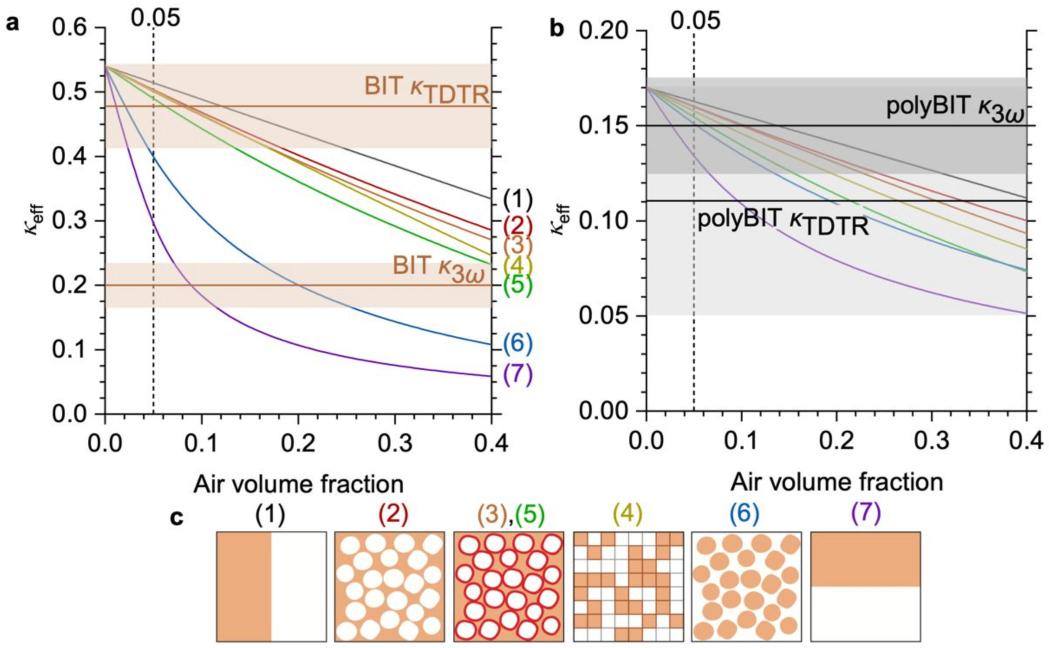

Figure S7: Effective medium model for (a) BIT and (b) polyBIT. The dashed line at V_{air} = 0.05 represents the estimated fraction of air present in the polycrystalline films, which was estimated using ImageJ software and a two-dimensional optical micrograph of BIT and polyBIT. The seven equations plotted here are presented in **Table S3**. Illustrations for each example are presented in orange, white, and red. Orange indicates the fraction of BIT material, white represents the fraction of air, and red represents a interfacial contribution between components.

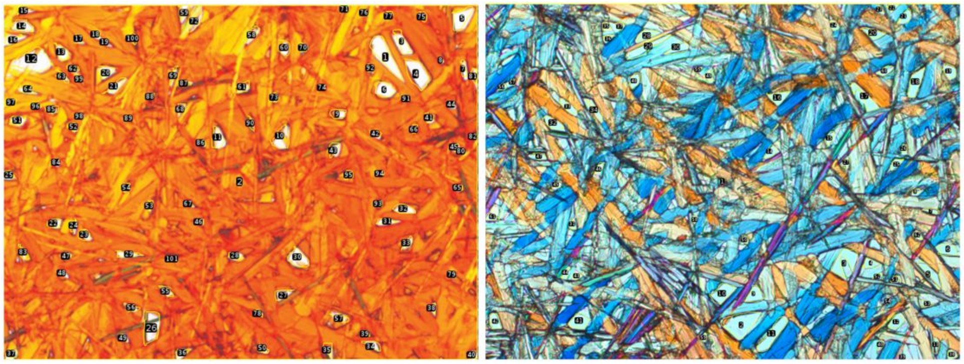

Figure S8: Polarized optical micrographs of BIT (left figure) and polyBIT (right figure) processed using ImageJ software to measure the total area of air voids.

4. Thermophysical characterization

4.1. Differential scanning calorimety and heat capacity at constant pressure measurements. Measurements were performed under N_2 atmosphere applying heating/cooling rates of 20 °C min^{-1} using a Mettler Toledo DSC3+. Thick films were scraped off glass substrates, placed in a MeltPrep Vacuum Compression Mold system, and compressed at room temperature using the Disc Tool into a disk to form better thermal contact between sample particulates and the Al pan. The mass of each disk was measured using the Mettler Toledo TGA/DSC 3+ to acquire an accuracy of 0.001 ± 0.0005 mg. The pans were crimped with a hole at the top of the Al lid to keep the pressure constant. For heat capacity measurements, Mettler Toledo's Alternating DSC (ADSC) method was employed. The method was programmed using a mean heating rate of 2 °C min^{-1}, a sinusoidal modulation of amplitude 2 °C, and a period of 1 min. A sapphire standard (SRM 720, NIST) was used to calibrate the measured c_p of the monomer and polymer. For conventional DSC runs, monomer, polymer, and empty pans were heated and cooled from 0 °C to 190 °C. For the monomer sample, a one-minute isotherm was programmed after the heating and cooling run to equilibrate the temperature of the pans. For the polymer sample, a 60-minute isotherm at 190 °C was programmed after heating run from 0 to 190 °C to de-polymerize the material.

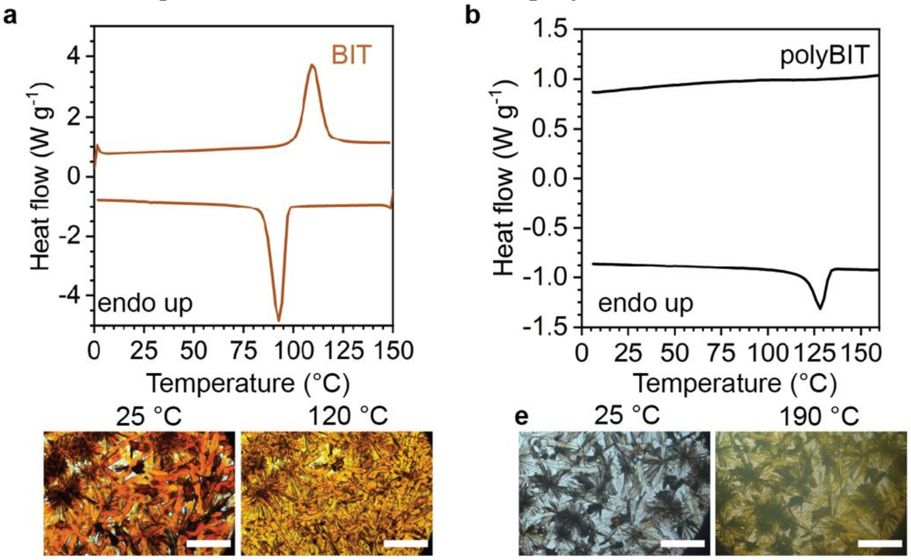

Figure S8: Heat capacity, calorimetry, and optical microscopy of BIT indicate a thermotropic transition is present in the monomer, suggesting these molecules have greater degrees of freedom to transport heat more efficiently. (a) Heat capacity at constant pressure of BIT and polyBIT. A latent heat contribution causes the heat capacity for BIT to increase more with temperature than polyBIT. This latent heat contribution is found in (b) the first heating and cooling cycle for thick BIT films. An endotherm and exotherm with onsets at approximately 100 °C indicate a thermotropic phase transition. This phase transition may accelerate degradation as seen in **Figure S8a**. (c) Corresponding optical micrographs for BIT before and just above 100 °C. Scale bar length represents 150 μm.

4.3. Thermogravimetric analysis. Degradation temperatures of monomer and polymer were acquired using Mettler Toledo TGA/DSC 3+. Thick films of the monomer and polymer were scraped off glass substrates and compacted into ceramic crucibles for thermal analysis.

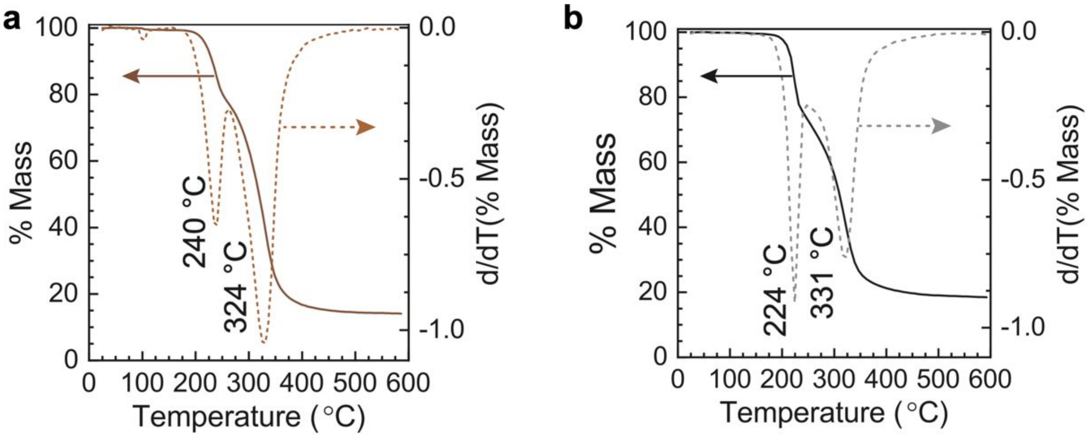

Figure S9: Minimal mass loss of BIT occurs below 200 °C before significant mass loss is measured at temperatures greater than 220 °C. Degradation profiles for a) BIT and b) polyBIT. The small degree of degradation below 200 °C can greatly affect the material's crystallization and the depolymerization yield.

It has been shown that polyBIT and similar derivatives can undergo the thermal depolymerization to convert back to BIT, but high monomer yields can only be achieved if the depolymerization reaction occurred in a solvent,[25] proving impractical for solid-state thermal switching.

5. References

1. L. Dou, Y. Zheng, X. Shen, G. Wu, K. Fields, W.-C. Hsu, H. Zhou, Y. Yang and F. Wudl, *Science*, 2014, **343**, 272-277.
2. S. D. Lubner, J. Choi, G. Wehmeyer, B. Waag, V. Mishra, H. Natesan, J. C. Bischof and C. Dames, *Review of Scientific Instruments*, 2015, **86**, 014905.
3. T. Yamane, N. Nagai, S.-I. Katayama and M. Todoki, *Journal of Applied Physics*, 2002, **91**, 9772.
4. Y. S. Ju and K. E. Goodson, *Journal of Applied Physics*, 1999, **85**, 7130-7134.
5. J. Liu, J. Zhu, M. Tian, X. Gu, A. Schmidt and R. Yang, *Review of Scientific Instruments*, 2013, **84**, 034902.
6. J. L. Braun, D. H. Olson, J. T. Gaskins and P. E. Hopkins, *Review of Scientific Instruments*, 2019, **90**.
7. D. H. Olson, J. L. Braun and P. E. Hopkins, *Journal of Applied Physics*, 2019, **126**.
8. C. Dames, *Annual Review of Heat Transfer*, 2013, **16**, 7-49.
9. T. Borca-Tasciuc, A. R. Kumar and G. Chen, *Review of Scientific Instruments*, 2001, **72**, 2139-2147.
10. S. Kommandur, Georgia Institute of Technology, 2019.
11. R. B. Wilson, B. A. Apgar, L. W. Martin and D. G. Cahill, *Opt. Express*, 2012, **20**, 28829-28838.
12. D. G. Cahill, *Review of Scientific Instruments*, 2004, **75**, 5119-5122.
13. A. J. Schmidt, X. Chen and G. Chen, *Review of Scientific Instruments*, 2008, **79**, 114902.
14. J. L. Braun, D. H. Olson, J. T. Gaskins and P. E. Hopkins, *Review of Scientific Instruments*, 2019, **90**, 024905.
15. H. Li, M. E. Decoster, C. Ming, M. Wang, Y. Chen, P. E. Hopkins, L. Chen and H. E. Katz, *Macromolecules*, 2019, **52**, 9804-9812.
16. D. G. Cahill and R. O. Pohl, *Solid State Communications*, 1989, **70**, 927-930.
17. G. Lv, E. Jensen, N. Shan, C. M. Evans and D. G. Cahill, *ACS Applied Polymer Materials*, 2021, **3**, 1555-1562.
18. J. M. Rinehart, J. R. Reynolds and S. K. Yee, *ACS Applied Polymer Materials*, 2022, **4**, 1218-1224.
19. R. Samanta, D. Kitagawa, A. Mondal, M. Bhattacharya, M. Annadhasan, S. Mondal, R. Chandrasekar, S. Kobatake and C. M. Reddy, *ACS Applied Materials & Interfaces*, 2020, **12**, 16856-16863.
20. X. Wang, V. Ho, R. A. Segalman and D. G. Cahill, *Macromolecules*, 2013, **46**, 4937-4943.
21. S. Spagnoli, J. Berrehar, J.-L. Fave and M. Schott, *Chemical Physics*, 2007, **333**, 254-264.
22. Z. Chen and C. Dames, *Applied Physics Letters*, 2015, **107**, 193104.
23. M. T. Agne, R. Hanus and G. J. Snyder, *Energy & Environmental Science*, 2018, **11**, 609-616.
24. Z. Chen, Z. Wei, Y. Chen and C. Dames, *Physical Review B*, 2013, **87**.
25. X. Luo, Z. Wei, B. Seo, Q. Hu, X. Wang, J. A. Romo, M. Jain, M. Cakmak, B. W. Boudouris, K. Zhao, J. Mei, B. M. Savoie and L. Dou, *Journal of the American Chemical Society*, 2022, **144**, 16588-16597.
